# From gut to mouth: salivary signatures of carcinogenic microbiota

**DOI:** 10.3389/fcimb.2026.1769355

**Published:** 2026-04-30

**Authors:** Fatima Zahra Kamal, Radu Lefter, Alin Ciobica, Vasile Burlui, Said Rammali, Achraf Abdou, Marius-Nicusor Grigore, Cătălina Ionescu, Daniela-Ivona Tomita, Mihaela Diana Gheban

**Affiliations:** 1 Care and Health Biology Team, 2S2D Laboratory, Higher Institute of Nursing Professions and Health Technical (ISPITS), Casablanca, Morocco; 2 Center of Antropological and Biomedical Research, Romanian Academy, Iasi, Romania; 3 Department of Biology, Faculty of Biology, Alexandru Ioan Cuza University of Iasi, Iasi, Romania; 4 ”Ioan Haulica” Institute, Apollonia University, Iasi, Romania; 5 ”Olga Necrasov” Center, Biomedical Research Group, Romanian Academy, Iasi, Romania; 6 Academy of Romanian Scientists, Str. Splaiul Independentei, Bucharest, Romania; 7 CENEMED Platform for Interdisciplinary Research, “Grigore T. Popa” University of Medicine and Pharmacy of Iasi, Iasi, Romania; 8 Clinical Department, Apollonia University, Iasi, Romania; 9 Human Nutrition, Bioactives and Oncogenetics Team, Faculty of Sciences, Moulay Ismail University, Meknes, Morocco; 10 Laboratory of Agro-Alimentary and Health, Faculty of Sciences and Techniques, Hassan First University of Settat, Settat, Morocco; 11 Laboratory of Organic Chemistry, Materials, Electrochemistry, and Environment, Faculty of Sciences Ain Chock, Hassan II University, Casablanca, Morocco; 12 Doctoral School of Biology, Alexandru Ioan Cuza University, Iasi, Romania

**Keywords:** early cancer detection, microbial dysbiosis, non-invasive screening, salivary diagnostics, tumor biomarkers

## Abstract

Recent oncological research has repositioned saliva as a non-invasive, dynamic diagnostic medium that reflects the molecular and microbial perturbations driving gastrointestinal malignancies. Growing evidence highlights characteristic salivary microbial fluctuations, such as the depletion of commensal species (*Neisseria elongata*, *Streptococcus mitis*) as well as enrichment of pathogenic taxa (*Granulicatella adiacens*, *Leptotrichia, Fusobacterium nucleatum*), which are distinctly associated with initiation of pancreatic, gastric, and colorectal cancers. Gastrointestinal cancers, on the other hand, alter salivary gland function and oral microbiota, increasing the risk of caries and periodontitis. Bacterial translocation via the oral-gut axis, a more common process than previously believed, appears to mediate this bidirectional relationship. Salivary circulating tumor DNA (ctDNA), microRNAs (miRNAs), extracellular vesicles (EVs), and proteomic signatures have established connections between oral and systemic health, and could be exploited for the detection and monitoring of oncogenic events. Challenges such as the low abundance of salivary biomarkers and the lack of standardized collection methods limit currently the application of saliva as a diagnostic tool, but it is expected next-generation sequencing, digital PCR, and nanoscale biosensors to pave the way toward precision salivary diagnostics.

## 1 Introduction

In recent years, cancer research has witnessed many advances in theory and practical clinical applications. One emerging and particularly compelling insight is the proposed relation between the individuals oral and systemic health (Okuyama and Yanamoto, 2024; Papale et al., 2022; Kapila, 2021). Many researchers now consider saliva as a factory of biochemical information that, with more in depth understanding and research insights, has the capacity to indicate very early stages of gastrointestinal (GI) cancers (Okuyama and Yanamoto, 2024; Papale et al., 2022; Kapila, 2021). Rather than serving solely as a digestive secretion, saliva is now recognized as a valuable, non-invasive diagnostic medium that harbors information related to an individual’s genetics and epigenetics, oral microflora, cellular activity, immune responses, and shifts in signaling patterns observed in a healthy state and during disease progression (Okuyama and Yanamoto, 2024; Papale et al., 2022; Kapila, 2021; Yoshizawa et al., 2013; Suragimath et al., 2024; Woźniak et al., 2019).

Many research studies have associated distinct microbial flora of the salivary gland and specific molecular signals with the increased risk of developing cancers of pancreas, gastric or colorectal origins (GI cancers) (Mishra et al., 2016; Schafer et al., 2014). Precisely, there is ample literary evidence that oral microflora influences the local (inflammatory) and systemic (immune) responses in individuals with GI cancers (Peng et al., 2022; Chandra Nayak et al., 2025). For instance, in pancreatic cancer, studies have reported a marked reduction in the colonization of *Neisseria elongata* and *Streptococcus mitis*, which are normal flora of the oral cavity (Farrell et al., 2012). These bacteria are increasingly replaced by pathogenic strains of *Granulicatella adiacens* and *Leptotrichia* sp. during disease progression (Farrell et al., 2012). Similarly, colonization of oral cavity by pathogens such as *Porphyromonas gingivalis* and *Fusobacterium nucleatum* are also reported in GI cancers (Durlak et al., 2025; Sun et al., 2020; Su et al., 2024; Karpiński, 2019). Unlike specific bacterial colonization in pancreatic cancer reported above, some other studies have reported non-specific microbial dysbiosis in GI cancers.

The microbial dysbiosis leads to several changes in the oral mucosa. They act as initiators of GI cancers, and also support the systemic progress towards carcinogenesis. For example, the pathogenic strains produce bioactive compounds and toxins, which accumulate in the mucosal tissues and compromise its immunity (Barbour et al., 2022; Culshaw et al., 2024; Kleinstein et al., 2020). It also influences host cell activity, inflammatory (NF-κB) signaling and immune responses. This is accompanied with upregulation of pro-inflammatory cytokines and chemokines. Altogether, these signals trigger a persistent inflammatory cascade causing oxidative stress, DNA damage and genomic instability (Durlak et al., 2025; Sun et al., 2020; Su et al., 2024; Karpiński, 2019). These pathogens then translocate to distant GI sites and disrupt the gut microbial balance, as reported in cases of colorectal adenocarcinoma and other GI tumors (Karpiński, 2019; Sun et al., 2020). On translocation, they trigger similar response in the GI tract. Altogether, these events create a micro-environment which is conducive to tumor initiation and progression.

The GI cancer’s conventional diagnosis is based on invasive tissue biopsies, endoscopic imaging and blood-based biomarkers (Yoshizawa et al., 2013; Suragimath et al., 2024; Woźniak et al., 2019). The proposed idea of using saliva as a non-invasive diagnostic tool can prove to be simple and effective, provided appropriate standardization protocols are developed to ensure results reproducibility (Yoshizawa et al., 2013; Suragimath et al., 2024; Woźniak et al., 2019). Saliva can provide a complete oral microbial fingerprint, and also indicate the biochemical changes that accompany the progression of a disease. For instance, the pancreatic and gastric tumors are associated with specific mutations which can be detected with the help of ctDNA analysis from saliva sample (Riviere et al., 2018; Abulsoud et al., 2023). The miRNA profiles highlight regulatory pathways modulations in cell proliferation, apoptosis, and immune activity (Riviere et al., 2018; Abulsoud et al., 2023). The genetic materials altered by tumor-derived signals are protected from enzymatic degradation in extracellular vesicles (EVs) in the oral cavity (Chiabotto et al., 2019; Zhan et al., 2020; Kosaka et al., 2019). Hence, they can also be used as an early indicator of GI cancers.

Salivary diagnostics has emerged as a promising, non-invasive method for the detection of cancer by employing various biomarkers, similar to blood biomarkers, making it suitable for rapid and accurate screening (Wang et al., 2017). A systematic review of salivary metabolites in cancer patients identified 140 distinct metabolites among 25 studies, with alanine, valine, and leucine being the most commonly reported (Assad et al., 2020). The review illustrated that specific metabolite combinations showed strong diagnostic performance, such as proline, threonine, and histidine for the diagnosis of breast cancer, and choline, betaine, pipecolinic acid, and L-carnitine for early detection of the oral cancer (Assad et al., 2020). Saliva’s diagnostic utility stems from its composition of blood-derived molecules that reflect systemic disease states, making sample collection simple, cost-effective, and patient-friendly (Zhang et al., 2012; Wang et al., 2016).

Recent progress in salivary diagnostics have made possible the high-resolution detection of carcinogenic microbial signatures and host-derived biomarkers linked to GI malignancies (Hu et al., 2025). Methods like 16S rRNA gene sequencing and shotgun metagenomics enable comprehensive analyze of salivary microbiota pattern associated with GI malignancy-related alterations in the microbiota. Meanwhile, salivary proteomics, metabolomics, and transcriptomics including exosomal microRNA profiling, help understand the tumor-induced molecular changes (Nijakowski et al., 2022). Incorporation of biosensor technologies and artificial intelligence, based analytical platforms further enhances saliva-based precision diagnostics potential for early detection, risk stratification, and disease monitoring in GI cancers (Mishra et al., 2016).

Though, saliva holds promise as a diagnostic tool, presently certain challenges limits its application in clinical practice (Chandrakala et al., 2024; Suragimath et al., 2024). These challenges include (i) low abundance of biomarkers (ii) variable collection methods (iii) biological factors such as age, inflammation, circadian variation and inter-individual differences and (iv) variable oral microbial flora (Li et al., 2024; Soares Nunes et al., 2015; Allaband et al., 2019). Some of these challenges (mainly low biomarker concentration and standardizing collection methods) can be overcome with the help of sophisticated analytical tools such as PCR, high-throughput sequencing, and nanoscale biosensors (Soda et al., 2019; Crosby et al., 2022). Many studies have reported high sensitivity and specificity of these methods (Munsif et al., 2025). However, standardizing biological factors and developing individual oral microbial fingerprints remains challenging (Gao et al., 2022; Brzychczy-Sroka et al., 2024; Rajasekaran et al., 2024). Standardizing these factors is necessary to ensure reproducibility of results across diverse patient populations. To a large extent, interdisciplinary researches in the fields of gastroenterology, oncology and dentistry can overcome the challenges related to biological factors and microbial fingerprint (Paqué et al., 2022; Guven, 2021). Hence, in near future, saliva-based monitoring can be expected to bring a paradigm shift and improve personalized medicine and early interventions (Paqué et al., 2022; Guven, 2021). Additionally, the interdisciplinary researches can provide better insights into the oral manifestations of GI malignancies, and identify the differentiating factors compared to periodontal disease or benign mucosal lesions (Rezasoltani et al., 2022; Boksa et al., 2025).

The present review aims to provide an overview of the challenges and progress in early and non-invasive saliva-based diagnosis of GI cancers. The study explores the common and distinguishing factors in oral diseases and GI cancers related to microbial dysbiosis and molecular signals.

## 2 Bacteria that link oral and GI cancers

### 2.1 Bacterial translocation from oral cavity to GI tract

As opposed to earlier belief that translocation of oral microflora to other sites in the human body is rare, recent studies have highlighted that bacterial translocation is a common process (Schmidt et al., 2018). In healthy individuals too, the oral microbial strains can be considered as a reservoir for GI tract (Schmidt et al., 2018). It is believed that the anatomy of human body connecting the oral cavity and GI tract along a single axis facilitates the microbial transmission. The physiological factors such as swallowing and movement of food along this axis further contribute to microbial translocation (Siddiqui et al., 2025; Yamazaki and Kamada, 2024). In fact, the process of swallowing approximately 1.5 liters of saliva daily serves as a persistent vehicle for translocating approximately 1.5 × 10^12^ oral bacteria into the GI tract. This process is further amplified by the continuous exposure to ingested foods and salivary secretions (Lu et al., 2022; Olsen and Yamazaki, 2019).

Beside the oral-gut axis, the hematogenous and lymphatic systems offer additional pathways for oral bacteria to enter the bloodstream or lymphatic system in cases of mucosal injury or inflammation. Subsequently, these bacteria travel to distal sites, including the gut (Kitamoto et al., 2020; Gendron et al., 2000). Some pathogens utilize the lymphatic system to reach organs or enter systemic circulation, causing more widespread infections and inflammation of the cardiovascular, respiratory, and GI systems (Acheson and Luccioli, 2004; Han and Wang, 2013). Only select sub-types of oral species appear prone to extra-oral translocation and pathogenesis (Han and Wang, 2013). Moreover, proton pump inhibitors, significantly increase this translocation by the gastric acid suppression and impairment of the barrier function, allowing elevated survival and transit of oral microbes into the distal gut (Zhang et al., 2024). These findings raise the need for further research to elucidate the molecular and environmental factors of oral-to-gut bacterial migration, as well as to design clinical interventions that could lessen the pathogenic colonization potential and its related health risk.

### 2.2 *Fusobacterium nucleatum* and their implications for GI cancers

*Fusobacterium nucleatum* is an oral anaerobic gram-negative bacterium that colonizes colon tissues by infecting bloodstream. This opportunistic pathogen has emerged as a significant etiological factor in colorectal cancer (CRC) (Li et al., 2022; Wang and Fang, 2023). In colon cancers, this pathogen is prevalent and commonly forms biofilms. Studies have indicated complex interactions of *F. nucleatum* with tumor microenvironment that affects local ecology (including virulence factors and intestinal metabolites) and tumorigenesis process (modulation of immune response and promotion of oncogenic microRNAs and DNA damage) in CRC (Wang and Fang, 2023; Wang et al., 2021; Li et al., 2022). Specifically, two essential virulence factors, FadA and Fap2, are identified that aid in intestinal epithelial cell adhesion (**Table 1**). These proteins are reported to cause immunosuppression by binding to immune cells (Sun et al., 2019). On binding, the bacterium enters the immune cells and activates TLR2/TLR4 signaling. This process initiates a pro-inflammatory cascade and creates a tumor microenvironment inside the immune cells (Sun et al., 2019). In addition, a complex molecular network formation (consisting of microRNAs and autophagy components) is reported on infection with *F. nucleatum*. This network is responsible for chemoresistance and tumor recurrence (Sun et al., 2019). Some data have further reported that specific subtypes of *F. nucleatum* are responsible for promoting tumor growth in CRC (Zepeda-Rivera et al., 2024; Bi et al., 2021). The colonization and proliferation of these specific subtypes are suggested to induce intestinal inflammation and promote CRC progression. These evidences strongly connect microbial dysbiosis to dietary patterns that induce a pro-carcinogenic environment (Lu et al., 2025). Altogether, the studies on *F. nucleatum* and its high-risk subtypes in GI tract advocate for improved microbial screening methods as a diagnostic strategy for patients with CRC. This strategy will be especially helpful for patients at elevated risk of CRC, or those receiving treatment for aggressive or resistant CRC (Ye et al., 2017). However, presently, we need more studies focused on understanding the interconnections between *F. nucleatum*, tumor biology and the gut ecosystem, to devise effective interventions to mitigate CRC progression (Li et al., 2022; Wang and Fang, 2023; Wang et al., 2021; Wu et al., 2019).

**Table 1:** Role of oral and gut-associated bacteria in gastrointestinal carcinogenesis.

Bacterial species	Primary oral reservoir	GI cancer association	Mechanisms implicated in carcinogenesis	Key references
*Fusobacterium nucleatum*	Periodontal pockets, tongue dorsum	Colorectal, pancreatic	FadA–E-cadherin binding, Fap2–TIGIT immune suppression, NF-κB activation, oncogenic miRNA induction, chemoresistance	Farrell et al., 2012; Sun et al., 2019; Wang et al., 2021; Li et al., 2022
*Porphyromonas gingivalis*	Subgingival plaque	Pancreatic, colorectal	Chronic inflammation, TLR2/4 activation, immune evasion, epithelial barrier disruption	Karpiński, 2019; Durlak et al., 2025
*Granulicatella adiacens*	Oral biofilm	Pancreatic	Replacement of commensals, pro-inflammatory microenvironment	Farrell et al., 2012; Mishra et al., 2016
*Leptotrichia* spp.	Oral mucosa	Pancreatic, colorectal	Acidogenic metabolism, mucosal barrier disruption, immune modulation	Farrell et al., 2012; Schafer et al., 2014
*Helicobacter pylori*	Oral cavity (secondary reservoir)	Gastric	CagA translocation, ROS/RNS production, DNA double-strand breaks, genomic instability	Hardbower et al., 2014; Kalisperati et al., 2017; Shimizu et al., 2017
*Streptococcus mitis*	Healthy oral flora	Protective (reduced in cancer)	Immune homeostasis, microbial balance	Farrell et al., 2012; Papale et al., 2022

### 2.3 *Helicobacter pylori* and their implications for GI cancer

*Helicobacter pylori* is a prevalent etiological agent associated with approximately 50% of patients with GI cancer. So far, *H. pylori* is the strongest known risk factor for GI cancer which employs multifaceted mechanisms to induce DNA damage and genetic instability. The pathogen is associated with modulation of molecular, cellular, as well as tissue-level signals, by inducing chronic inflammation and oxidative stress (Hardbower et al., 2014; Kalisperati et al., 2017). In fact, production of Reactive Oxygen Species (ROS) and Reactive Nitrogen Species (RNS) are the key bacterial mechanisms that induce significant oxidative stress in the gastric mucosa and progresses towards carcinogenesis (Handa et al., 2010). The oxidative stress is primarily mediated through activated neutrophils in GI tract, though *H. pylori* is also known to generate ROS (Handa et al., 2010, 2011). The chronic oxidative stress consequently results in a chronic inflammatory state, leading to extensive DNA damage in the gastric epithelial cells. The double strand breaks are also commonly reported that promotes genomic instability and neoplastic transformation (Butcher et al., 2017; Kalisperati et al., 2017; Shimizu et al., 2017). The compromised GI health subsequently limits DNA damage repair machinery and increases activation-induced cytidine deaminase levels; thereby affecting genome integrity. Thus, at a molecular level, the *H. pylori* infection first induces mutagenesis, which then progresses to tumorigenesis (Touati, 2010).

*H. pylori* cells transfer CagA oncoprotein into gastric epithelial cells through specific host cell surface (α5β1 integrin) receptors and type IV secretion system. The bacterium binds to integrin α5β1 receptors using the CagL adhesin protein, containing arginine-glycine-aspartate motif. These motifs aid in binding and activating the integrins (Kwok et al., 2007; Backert and Selbach, 2008). *H. pylori* further induces externalization of phosphatidylserine, present typically on the inside of the host plasma membrane, to facilitate cellular entry of CagA and its membrane localization (**Table 1** (Murata-Kamiya et al., 2010). This process is different from conventional endocytic pathways and depends on cellular mechanisms for energy requirements (Murata-Kamiya et al., 2010). On internalization, CagA is phosphorylated at the tyrosine residue by Src family kinases. This severely disrupts the normal cellular processes such as rearrangement of actin cytoskeletal, cell-to-cell junctions, and regulation of cellular polarity. Altogether, these disrupted cellular processes lead to initiation of cancer (Backert and Selbach, 2008; Murata-Kamiya et al., 2010).

The pathogen-encoded virulence factors, along with altered cellular and molecular signals, which occur on colonization of GI tract with *H. pylori* bacteria activate the inflammatory and immune responses in the mucosa. These multiple events occurring simultaneously severely compromises host immunity and GI health and weakens cellular repair mechanisms, and thus increases susceptibility to carcinogenic events (Hardbower et al., 2014; Shimizu et al., 2017). Further than gastric pathology, *H. pylori* colonization significantly influences oral health. A study on oral colonization of *H. pylori* and GI disease correlation reported presence of *H. pylori* in 40-54% of examined individuals in their study, with higher rates observed in patients with periodontal diseases (Jun et al., 2011; Czesnikiewicz-Guzik et al., 2005). *H. pylori* infection has a positive correlation with various oral diseases such as dental caries, periodontitis, and oral lichen planus (Fan et al., 2025). The bacterium modulates the diversity and structure of the oral microflora by its interactions with oral pathogens such as *Streptococcus*, *Porphyromonas gingivalis* and *Candida albicans* (**Table 1**) (Fan et al., 2025). The oral cavity serves as a potential reservoir for gastric infection and re-infection, with approximately 13% annual recurrence rates attributed to oral *H. pylori* colonization (Yee, 2016). However, oral *H. pylori* alone do not significantly affect hormones like ghrelin and gastrin, unlike gastric infections (Czesnikiewicz-Guzik et al., 2005).

## 3 Cancer-associated microbiota impact on saliva composition and function

### 3.1 pH and buffering changes

Bacteria related GI cancers associated with *F. nucleatum* and *H. pylori* can influence physicochemical properties of saliva, especially its pH and buffering ability (Hara et al., 2024; Kageyama et al., 2019). Normally, the phosphate and bicarbonate ions present in the saliva provides buffering action, which helps in maintaining its pH despite production of acids during metabolic activities of oral and systemic micro-organisms (Lynge Pedersen and Belstrøm, 2019). Saliva also maintains the population of microbial communities under control with the help of proteins such as lysozymes, mucins, lactoferrins, and histatins (Lynge Pedersen and Belstrøm, 2019). Oncogenic bacterial colonization in the oral cavity alters local inflammatory responses and metabolic outputs, leading to reduced bicarbonate concentrations and impaired phosphate balance, which collectively contribute to an acidic oral microenvironment (Wang et al., 2024c; Meurman, 2010; Pignatelli et al., 2022). This acidic environment facilitates cariogenic activity and is also thought to act as a signal of systemic dysbiosis associated with tumorigenesis (Wang et al., 2024a; Fitzsimonds et al., 2020). Thus, altered pH of saliva may be an indirect manifestation of changes occurring in the oral–gut microbial axis that may lead to initiation of GI cancers.

### 3.2 Immune proteins

The overgrowth of microbes in the salivary gland is prevented with the help of immune proteins that act as antimicrobial components. Few examples include secretory immunoglobulin A (SIgA), mucins, lactoferrins, lysozymes, and histatins. These compounds either inhibit the bacterial growth directly or prevent their adherence to oral mucosa (Lynge Pedersen and Belstrøm, 2019; Marcotte and Lavoie, 1998). However, colonization of salivary glands and oral cavity by specific microbes associated with cancer alter the level of these proteins (Cultrera et al., 2025). Oncogenic bacteria such as *F. nucleatum* or *H. pylori* either suppress or elevate the salivary inflammatory cytokines allowing persistence of bacteria or eliciting a chronic inflammatory response, respectively (Rai et al., 2021; McIlvanna et al., 2021; Karpiński, 2019; Whitmore and Lamont, 2014). For instance, the reduction in IgA levels impair mucosal immunity and leaves it vulnerable to infection during microbial translocation from the mouth to the gut. The altered lactoferrin levels is indicative of the host cellular defense mechanism to prevent iron acquisition by the pathogen (Kulkarni and Ruprecht, 2017; Fagarasan and Honjo, 2004; Lynge Pedersen and Belstrøm, 2019; Valenti and Antonini, 2005). Thus, it is clear that oral and GI tract bacteria associated with cancer play a role in actively modulating salivary immune defenses, which in turn influence oral microflora and increase the risk of cancer (Cosseau et al., 2008; Cultrera et al., 2025).

### 3.3 Proteomic and metabolomic alterations detectable in cancer patients

The diagnostics have advanced suitably in the recent years with the help of metabolomic and proteomic analysis of blood and body fluids. These diagnostic tools have identified some modifications associated with cancers using saliva as a sample (Nijakowski et al., 2022; Winck et al., 2015; Bartlett et al., 2020; Zhang et al., 2010b; Streckfus and Dubinsky, 2007). It is very likely, that these modifications are initiated, or partly driven, by the interactions occurring between the oral and GI tract bacteria. The metabolomic and proteomic profile of saliva show changes in the level of cytokines, antimicrobial peptides, and tumor-associated proteins when oncogenic microbes colonize the intestine (Sjögren et al., 2009; Roi et al., 2020; Zong et al., 2020; Gallo et al., 2024; Zitvogel et al., 2024; Medeiros et al., 2023; Bartlett et al., 2020). In the process, they alter host metabolism, immune signaling, and oxidative stress (Rai et al., 2021; Wang et al., 2017). Additionally, they alter the level of amino acids, polyamines, and lipid derivatives. Many of these changes are associated with tumor metabolism (Johnson et al., 2016; Yang et al., 2019; Smith et al., 2022; Al-Ansari et al., 2021; Liu et al., 2024; Wikoff et al., 2009). Overall, these findings collectively provide useful insights into the disruption mechanisms followed by *F. nucleatum* and *H. pylori*, and further support the promising potential of metabolomic and proteomic profile of saliva as a diagnostic aid in detecting GI cancers at an early stage.

## 4 Gastrointestinal cancers impact on oral health

### 4.1 Oral symptoms associated with GI cancers

Many symptoms of oral diseases overlap with those of GI cancers. These symptoms affect the function as well as appearance of soft and hard tissues, which can be easily observed before disease progression to advanced stages, as noted during a case of inflammatory bowel disease (Al-Zahrani et al., 2021; Logan, 2010). Hence, early recognition is possible with suitable interventions, which can ensure timely diagnosis and treatment of disease (Park et al., 2024; Daley and Armstrong, 2007; Logan, 2010; Ansari et al., 2025). For example, CRC has been associated with distinctive vascular alterations in the oral mucosa, which may be identified during oral examinations and could prompt further GI evaluation if detected alongside other risk factors (Latini et al., 2012; Roy, 2003; Logan, 2010). According to Latini et al., alterations in the color of the oral mucosa can be used as a clinical biomarker for cancer detection. CRC patients exhibit 96–99% diagnostic accuracy, with much lower red and green values, and greater blue values, than healthy controls (Latini et al., 2012). Roy (2003) particularly observed changes in the buccal and subgingival mucosa vascular patterns in patients with hereditary non-polyposis CRC, indicating that these changes can be a reflection of subclinical extracellular matrix abnormalities that contribute to the development of cancer (Roy, 2003). Furthermore, Janati et al. examined data that connected bacterial infection, inflammation, and nutrition to CRC through the processes of chronic dental diseases such periodontitis and tooth loss (Idrissi Janati et al., 2016). Since the above mentioned oral manifestations often precede systemic signs and symptoms, dental professionals play a major role in detecting these early indicators. Also, managing patients with GI diseases requires careful consideration of bleeding risks, infection susceptibility, and malnutrition status (Al-Zahrani et al., 2021). Hence, with a multidisciplinary research approach to improve our current understanding of the associations between oral and GI health, it is possible to improve patient care and treatment outcomes (Logan, 2010).

The reported prevalence of Metastatic Colon Adenocarcinoma (MCA) initiating from oral dysbiosis is rare, and accounts for only 1% of all oral malignant tumors (Neumann et al., 2021; Baranovic et al., 2015). These incidences are reported to occur in the gingiva and maxillary palate in the form of pedunculated nodules, swellings, or masses that appear as benign lesions (De Almeida Lança et al., 2023; Dalirsani et al., 2020). Few examples of such cases have been reported in the literature. One review documented 45 clinical cases spanning several decades, only two of which clearly involved the palate (De Almeida Lança et al., 2023). Another review collated research studies from previous 3 decades and reported 27 cases (Neumann et al., 2021). The diagnosis of MCA is confirmed based on the presence of CK20 on immune-histochemical analysis, and appearance of columnar epithelium with papillary areas on histopathological examination of the biopsy samples (De Almeida Lança et al., 2023). In MCA, the presentation of oral metastases indicates advanced disease and a poor prognosis (Dalirsani et al., 2020). Hence, the appearance of oral lesions should be reconsidered as a possible differential diagnosis for MCA. Also, conversely, patients with a history of adenocarcinomas should be thoroughly examined for presence of oral lesions to ensure proper disease prognosis (Baranovic et al., 2015; Dalirsani et al., 2020).

Unfortunately, complications also arise in differential diagnosis between GI cancers and oral diseases due to similar manifestations presented by other GI conditions such as gastro-esophageal reflux disease (GERD). GERD also shows oral symptoms such as burning mucosal sensation, tooth erosion, halitosis, xerostomia, and mucosal erythema. Although this complicates disease diagnosis, it strongly suggests a connection between systemic GI conditions and oral health (Di Fede et al., 2008; Preetha et al., 2015). Other oral symptoms of GI conditions include mucositis, aphthous-like ulcerations, dysgeusia, and gingivitis. In GERD, gingivitis is worsened by gastric acid irritation due to dysfunction of esophageal sphincter and reduction in the flow of saliva (Mahajan et al., 2022; Memè et al., 2024). As a result the stomach acids can travel upwards and harm the oral mucosa. It is further noted that the severity of GERD is inversely proportional to the pH of saliva (Preetha et al., 2015; Memè et al., 2024).

### 4.2 Chemotherapy and radiotherapy for GI cancers contribute to oral health complications

Chemotherapy and radiotherapy, the two main GI cancer treatments, contribute to a wide range of oral health issues owing to their lack of selectivity and cytotoxic effects on both tumor as well as normal rapidly multiplying cells and tissues of oral and GI mucosa (Jones and Rankin, 2012; Chaveli-Lopez, 2014). Up to 70% of cancer patients undergo chemotherapy, and among them, approximately 40% experience oral complications arising from direct or indirect stomatotoxic effects. The most common oral sequelae include xerostomia, resulting from salivary gland mucositis, characterized by inflammation and ulceration of oral mucosa, and infections due to loss of mucosal integrity (Jones and Rankin, 2012; Wong, 2014). Other complications include osteonecrosis, dental alterations, neurological disorders, dysgeusia, and bleeding tendencies (Chaveli-Lopez, 2014). Since, some of these complications can be life-threatening, or significantly affect the patient’s quality of life, it highlights the importance of preventive oral health management (Wong, 2014; Chaveli-Lopez, 2014).

### 4.3 Gastrointestinal cancers and their impact on the oral environment and caries susceptibility

**Figure 1:**
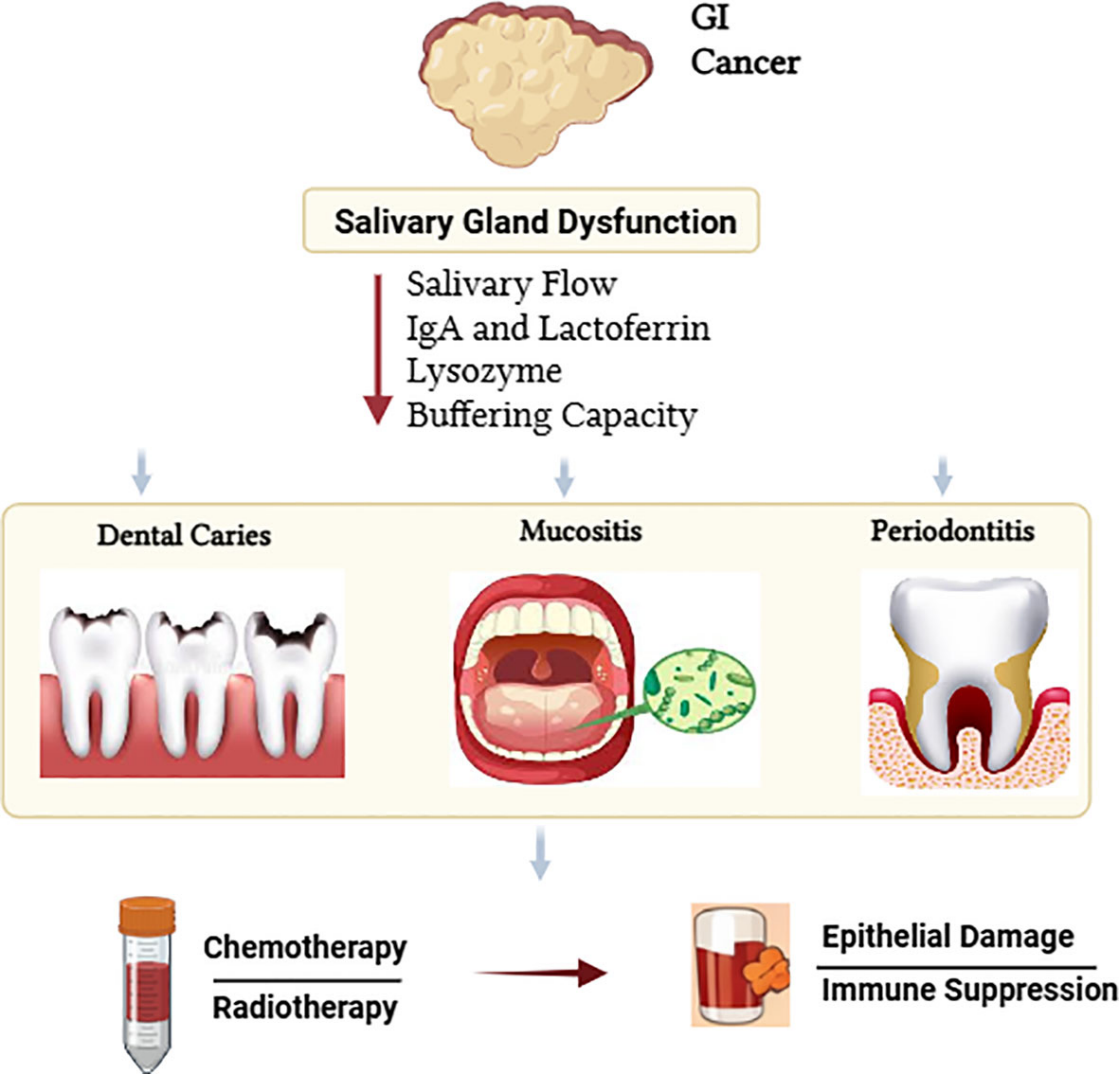
Impact of gastrointestinal (GI) cancers on oral health.

The alterations occurring in the oral microflora during the progression of GI cancers cause significant changes in the oral environment (**Figure 1**). Alongside, many changes occur due to primary immune responses and cancer-related treatments. The complex interactions between systemic diseases and these factors increase susceptibility to dental caries (Knop-Chodyła et al., 2024; Zhang et al., 2019). Also, periodontitis and gingivitis are common symptoms in oral cancers and malignancies of esophagus, stomach, and pancreas. Altogether, these commonalities suggest a bidirectional relation between oral and GI health (Madsen, 2021; Knop-Chodyła et al., 2024). In GI cancers, the production of pro-inflammatory cytokines, induction of abnormal immune responses and disruption of cellular metabolism promotes carcinogenesis and also disrupts normal oral microflora by supporting colonization of pathogens (Knop-Chodyła et al., 2024; Huang et al., 2025). These pathogens are recognized as stubborn due to formation of biofilms and enhanced acid producing ability. As a result they accelerate the development of dental caries (Marsh, 2018; Spatafora et al., 2024; Almståhl et al., 2018; Ruan et al., 2025; Rizzato et al., 2019). At the same time, impaired defense mechanisms of the salivary glands during GI cancers further increases the patients susceptibility to secondary caries (Sun et al., 2016; Lynge Pedersen and Belstrøm, 2019).

As indicated earlier in this review, saliva is responsible for maintaining the normal microflora of the oral cavity. This is achieved with the help of sIgA, lactoferrin, lysozyme, and mucins (**Figure 1**) (Lynge Pedersen and Belstrøm, 2019). The immunity of oral mucosa, however, weakens with progression of GI cancers and their treatments, due to reduction in sIgA levels and altered levels of lysozyme (Sun et al., 2016). The impaired immunity and weakened functions of salivary gland further reduces its buffering capacity, microbial homeostasis, and enhances demineralization (Jensen and Pedersen, 2016; Lynge Pedersen and Belstrøm, 2019). In such a scenario, caries remains a major issue in GI cancers due to reasons such as cancer-related dietary modifications, which happens due to increased cellular metabolic activities, appetite loss, or side effects associated with cancer treatments. These factors increase retention of fermentable carbohydrates in the oral cavity, which increases bacterial acidogenesis leading to dental caries (Al-Dakkak, 2011; Meurman and Grönroos, 2010; Levi and Lalla, 2018; Hong et al., 2010; Holma et al., 2020; Featherstone, 2000).

The host capacity to maintain a normal population of oral microbial flora further diminishes in GI cancers due to systemic immunosuppression that happens due to the malignancy itself or chemotherapy. This increases the chances of oral infections and diseases (Sixou et al., 1996; Epstein and Chow, 1999; Epstein, 2007; Soutome et al., 2021; Hong et al., 2010). Besides, behavioral risk factors including smoking, alcohol use, poor dietary habits, and ongoing or persistently recurring microbial infections predisposes an individual with or without GI cancers to an increased risk of dental caries (Soutome et al., 2021). Moreover, cancer therapies exacerbate oral complications, including xerostomia, mucosal atrophy, and long-term salivary gland dysfunction, all of which accelerate caries progression (Wong, 2014; Jensen and Saunders, 2021).

In summary, so far it is understood that events including salivary dysfunctions, dietary changes, and systemic immune deficiencies, that occur during GI cancers increase the risk of secondary dental caries. To better our understanding and overcome the challenges associated with these events, a possible approach may be based on integrating dentistry and oncology treatment plans. Precisely, we need strategies that can enable early diagnosis of oral diseases and a regularly updated profile of oral microbiome, along with personalized prevention strategies to reduce the risk of dental caries. Achieving this strategy may provide us with the required control that may help in reducing the systemic burden of GI cancers.

### 4.4 Relation between GI cancers and periodontal inflammation

**Figure 2:**
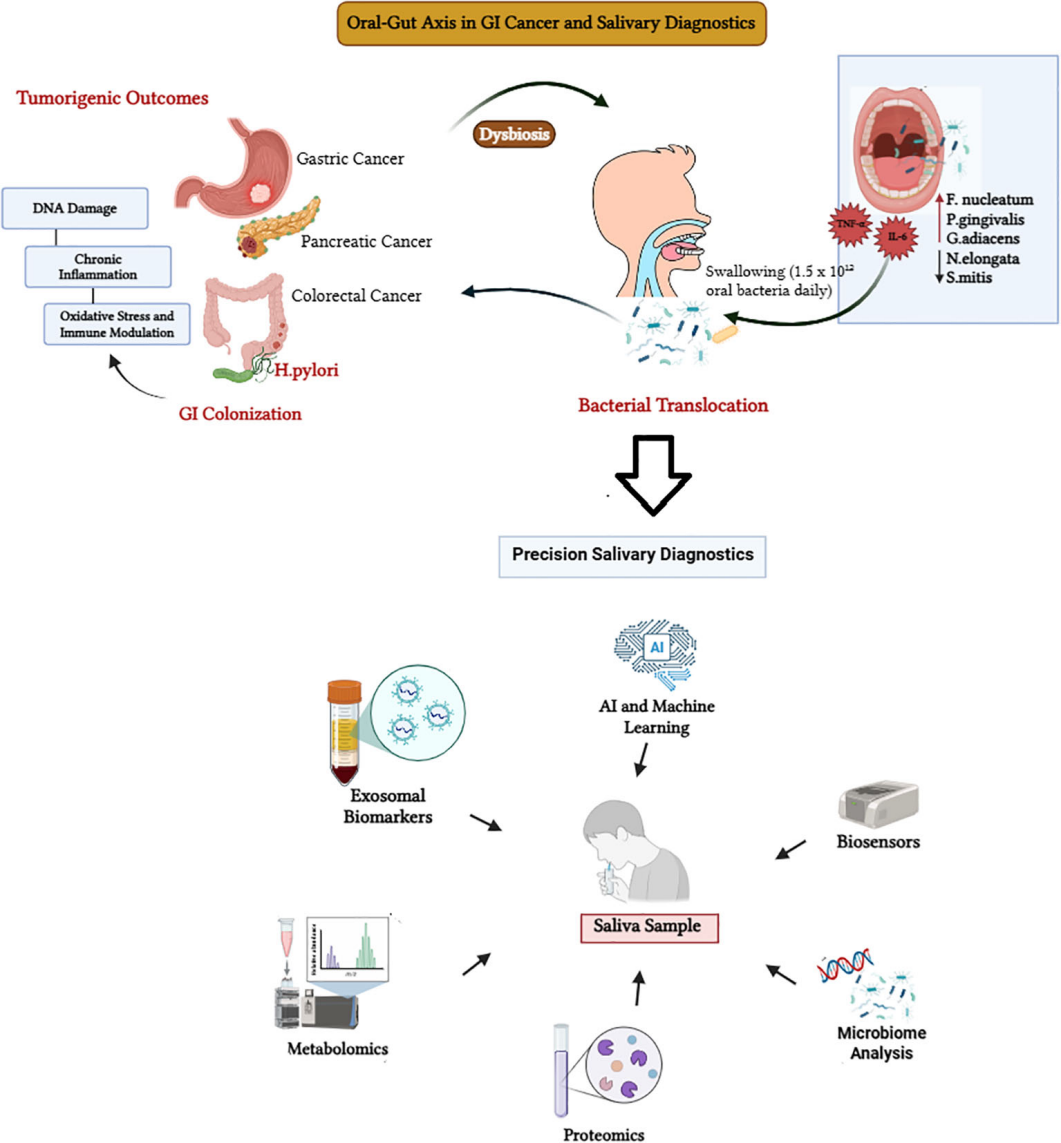
Oral-Gut microbial axis in gastrointestinal (GI) carcinogenesis and precision salivary diagnostics.

Both biological and psychosocial factors appear to drive the observed association between periodontal disease and gastrointestinal (GI) cancers (Wang et al., 2024b). Periodontitis is frequently observed in patients with GI malignancies and is characterized by gingival inflammation and progressive periodontal tissue destruction. Chronic systemic inflammation, cancer-associated immune modulation, and gut microbial dysbiosis are believed to increase susceptibility to periodontitis in this population (Wang et al., 2024b) (**Figure 2**). However, the reverse relationship –namely, the prevalence and characteristics of periodontitis among patients with established GI cancers --remains insufficiently explored in the current literature (Wang et al., 2024b; Zhang et al., 2020; Kulić et al., 2025; Lee and Choi, 2013). The cancer treatments also contribute to accelerated tissue degeneration and periodontal inflammation by disrupting microbial and immune balance (Novaes et al., 2022; Gusman et al., 2019). During cancer treatment with chemotherapy and radiotherapy, the pathogenic bacteria persist due to suppressed immune responses and further contribute to inflammation (Jin et al., 2025; Soni, 2017). The psychological stress associated with cancer diagnosis and treatment also increases the severity of periodontal disease, by influencing inflammatory pathways. The severity of periodontitis is reported to be independent of salivary flow or nutrition (Bakri et al., 2013; Bangalore Varadhan et al., 2019; Rai et al., 2011; Silva et al., 2024). These insights also suggest that integrating oncology, dentistry and psychological health management may result in improved GI cancer treatment outcomes.

There is strong evidence connecting microbial translocation, immune regulation, and chronic inflammation with GI cancers and periodontitis (**Figure 2**) (Baima et al., 2023). Studies have suggested that oral pathogens such as *F. nucleatum* migrate from the periodontium to GI tract, especially during immunosuppression or chemotherapy treatments in GI cancers (Akbari et al., 2024; Baima et al., 2023). After migration, they colonize the mucosa, causing changes in the epithelial barrier. In the process, they trigger chronic inflammation and disrupt immune signaling pathways, and initiate tumor development and spread of malignancies (**Figure 2**) (Baima et al., 2023; Akbari et al., 2024; Wu et al., 2019; Pignatelli et al., 2023). In absence of GI cancers, the oral microbiome dysbiosis in periodontitis alters the level of cytokine, chemokine, and growth factors (Santacroce et al., 2023; Hajishengallis, 2015), and impairs immunity. Thus, the dysbiosis in presence or absence of GI cancer is interconnected. They create a pro-inflammatory cascade that impairs antitumor immunity, which prevents detection of malignant cells (Pai et al., 2023; Elebyary et al., 2021). Despite the established associations, the likelihood of developing periodontitis in GI cancers, compared to general population, is presently unclear. This is because most of the available research describes the higher likelihood of developing GI cancers in patients with established periodontitis. Literature rarely discusses the former scenario or examines the prevalence of periodontitis among patients with GI cancers (Wang et al., 2024; Zhang et al., 2020). This wide research gap highlights the need for well-designed and multi-centric studies that compare the periodontal health of patients with GI cancer and healthy individuals. Such studies will help in understanding the associations between periodontitis and GI cancers, identify common risk factors, and develop preventive strategies for both oral and systemic diseases.

## 5 Saliva as a non-invasive biomarker: advances in salivary biosensors and point-of-care devices

### 5.1 Decoding digestive tumors through salivary molecular fingerprints

Encouragingly, GI cancer- related proteins are distinctly identified in human saliva highlighting their potential as non-invasive diagnostic indicators. Hence, the molecular profile of saliva can be exploited for detection and monitoring of GI cancers through specific biomarkers across proteomic, genomic, transcriptomic and metabolic categories (Khan et al., 2024; Xiao et al., 2016; Zúñiga-Pérez et al., 2024). A previous study identified 48 differentially expressed proteins in the saliva of gastric cancer patients using tandem mass spectrometry tag quantification (Xiao et al., 2016). Specifically, cystatin B (CSTB), triosephosphate isomerase (TPI1), and deleted in malignant brain tumor 1 (DMBT1) proteins showed sensitivity and specificity of 85% and 80%, respectively, indicating their exceptional diagnostic value (Xiao et al., 2016). A recent systematic review also reported the diagnostic accuracy of these proteins for GI cancer (Zúñiga-Pérez et al., 2024).

Seven distinct upregulated mRNAs have been detected in the saliva of patients with oral squamous cell carcinoma (OSCC), a type of GI cancer, indicating the suitability of mRNA profiling for its detection (Aro et al., 2019; Yakob et al., 2014). Other studies have reported detection of circulating tumor DNA in saliva of patients with GI cancers, and indicated its validity in early diagnosis and improved treatment outcomes (Kaczor-Urbanowicz et al., 2019; Kaczor-Urbanowicz et al., 2017; Cristaldi et al., 2019). These studies reporting association of specific proteins, mRNAs, and tumor DNA with cancer types, convincingly provide evidence that saliva may be a valuable diagnostic tool for early cancer diagnosis, and differential cancer diagnosis in some cases. However, before recognizing saliva as a suitable diagnostic tool, universally adopted validation and standardized methods is necessary (Zhang et al., 2016; Aro et al., 2019). Precisely, we need refined detection methods, suitable controls for confounding factors, and integrated multi-analyte panels to guarantee reliable and reproducible results.

### 5.2 Salivary biomarkers in digestive oncology

The studies documented so far strongly indicate the strong potential of salivary biomarkers in diagnosis of GI cancers, due to their enhanced sensitivity and specificity compared to traditional blood tests (Mishra et al., 2016; Wang et al., 2017). For instance, a meta-analysis study reported 85% (n=155 units) accuracy, and sensitivity and specificity of 0.76 each, of salivary biomarkers in detecting non-oral malignant tumors (Rapado-González et al., 2020). Moreover, the ability of these biomarkers in differential diagnosis of cancer types, including pancreatic, breast, lung, and gastric cancers has gained significant attention of researchers (Ghosh et al., 2024). For example, a study reported that four messenger RNA biomarkers including KRAS, MBD3L2, ACRV1, and DPM1, in the saliva, can potentially distinguish pancreatic cancer from other cancer types with a sensitivity and specificity of 90.0% and 95.0%, respectively (**Table 2**) (Zhang et al., 2010a). Differential diagnosis of pancreatic cancer with 92% accuracy has been reported by Liu et al. (2017) with the help of 29 novel salivary biomarkers. More recently, Al Balushi et al. (2024) suggested highly sensitive and specific polyamines and long noncoding RNAs (HOTAIR and PVT1) in saliva as promising alternatives to serum biomarkers. Similarly, the feasibility and suitability of saliva-based diagnostics has been reported in identifying pancreatic ductal adenocarcinoma (Tiffon, 2020).Compared to existing diagnostic practices, the biomarkers in saliva are reported to have higher specificity. This considerably minimizes false-positive outcomes and the need for unnecessary interventions (Surdu et al., 2025; Chandrakala et al., 2024). With advantages such as simplicity, non-invasive approach and feasibility, many studies have strongly advocated the use of saliva in GI cancer diagnosis. The clinicians prefer this diagnostic approach for ease of disease monitoring, while patients can undeniably advantage from the resulting comfort and compliance (Kausar et al., 2025). Thus, use of saliva in diagnosis of GI cancers holds transformative potential (Rashid et al., 2024; Wang et al., 2017; Yoshizawa et al., 2013). The current challenges faced in standardization of salivary diagnostics for rapid and highly accurate GI cancer detection includes rigorous validation, standardized collection protocols, and its integration with existing diagnostic systems (Mishra et al., 2016). To a large extent, these can be overcome with the help of biosensor and bioelectronic devices discussed in the next section.

**Table 2:** Clinical and preclinical studies evaluating salivary diagnostics in gastrointestinal cancers.

Cancer type	Salivary biomarkers	Study design	Key findings	References
Pancreatic	mRNA (KRAS, MBD3L2, ACRV1, DPM1)	Case–control	Sensitivity 90%, specificity 95%	Zhang et al., 2010
Pancreatic	miRNAs, polyamines	Systematic review	High diagnostic accuracy in early-stage disease	Al Balushi et al., 2024
Gastric	Proteins (CSTB, TPI1, DMBT1)	Proteomics validation	Sensitivity 85%, specificity 80%	Xiao et al., 2016
Colorectal	ctDNA, microbial signatures	Observational	Detectable tumor-derived DNA in saliva	Kaczor-Urbanowicz et al., 2019
Multiple GI	EV-derived miRNAs	Pilot clinical trial	Tumor-specific EV enrichment	Chiabotto et al., 2019; Zhan et al., 2020

### 5.3 Recent advancements in point-of-care devices utilizing saliva

Currently, the accessibility and diagnostic accuracy of saliva based Point-Of-Care (POC) devices have been improved with the help of nanoparticle biosensor technology, which integrates microfluidics, paper-based technologies, and smartphone-based platforms (Zarei, 2017; Moulahoum et al., 2023; Tavakoli et al., 2022; Khan et al., 2017). The microfluidic devices eliminate the need for large, complex laboratory equipment by enabling automated liquid handling in micro-sized devices. It also allows development of cost-effective and disposable tests that can be performed easily at home or in a clinical setting (Khosla et al., 2022; Weigl et al., 2008; Hu et al., 2017). Additionally, the microfluidic systems are valuable in diagnostics since they facilitate the pre-conditioning of complex saliva samples, which is critical for effective POC testing. They also reduce human error related to fluid transfer, and thus improve reliability (Herr et al., 2007). Beside microfluid system, the convergence of biosensors with mobile devices has enabled rapid, on-site detection of different biomarkers including proteins, peptides, enzymes, antibodies, and nucleic acids in saliva. These biomarkers can be used for monitoring cancers, infectious diseases like COVID-19 and other chronic diseases (Senf et al., 2020; Klebes et al., 2024; Kumari et al., 2024). The technological advancements have considerably aided in developing non-invasive, sensitive, specific and cost-effective diagnostic tools, and enabled personalized health monitoring and early disease intervention (Wong, 2006). Hence, we have already taken a major innovative leap in diagnostic procedures. To ensure consistent performance across varied sample populations, we are now required to develop protocols for user-friendly sample collection along with robust and standardized data interpretation.

## 6 Clinical challenges and future prospects of salivary diagnostics in digestive oncology

### 6.1 Analytical and biological challenges

The advances in diagnostic approaches using saliva have great potential in detecting cancers and other chronic diseases. However, some challenges remain in its application (Pfaffe et al., 2011; d’Amone et al., 2021). Among these, the low abundance of target molecules in saliva as compared to blood is a major analytical challenge. Hence, although changes may have occurred in the biochemistry of saliva during disease initiation and progression, early diagnosis may be difficult, and require extremely sensitive analytical tools (Soares Nunes et al., 2015; Pfaffe et al., 2011). At the same time, variations occur in saliva composition depending on the collection method used, timing of sample collection, and patient-specific factors. These challenges hinders development of reliable reference intervals, which are necessary for reproducibility of results and reliable data interpretation (Wang et al., 2015; d’Amone et al., 2021; Aps and Martens, 2005; Zhu et al., 2020).

The diagnostic reliability of saliva is further complicated by variable expression profiles of miRNA among individuals, due to influence of biological factors such as age and inflammation (Witwer, 2015). Also, presently, miR-191 and miR-16 are commonly used endogenous and exogenous miRNA controls, respectively. However, miRNA normalization data currently requires thorough validation in larger, more varied cohorts (Meyer et al., 2010; Peltier and Latham, 2008; McDermott et al., 2013). Similarly, leveraging EVs as diagnostic tools is constrained by difficulties in distinguishing tumor-derived EVs from those originating from normal cells. This necessitates the development of highly specific and stable markers for effective enrichment (Saleem et al., 2022).

### 6.2 Distinguishing cancer-related oral signs

Oral GI cancer manifestations can be subtle and often similar to conditions commonly seen in the oral diseases, creating an added challenge to differential diagnosis (Al-Zahrani et al., 2021). For instance, symptoms such as Red or white patches, trismus, reduced tongue mobility, and referred pain are common in primary oral diseases, metastatic lesions as well as paraneoplastic phenomena (Beddis et al., 2014; Parmar and Brown, 2020; Garnett et al., 2008; Malek and Damian, 2018; Ihde, 1987; Brady, 1996). Single or clusters of symptoms are generally poor discriminators of the underlying malignancy of GI, often necessitating further investigations when empirical treatment is ineffective (Logan, 2010; Hodgson et al., 2006; Jajam et al., 2017). Since, early manifestations of chronic diseases such as GI cancers are often easily missed, and alarming features do not appear until latter stages of disease, improved outcomes can be achieved only through collaborations between gastroenterologists, dental professionals, and oncologists.

### 6.3 Future opportunities in salivary diagnostics

The existing limitations may appear vast at present, but the scope of emerging technologies are wider and offer significant potential to overcome the current obstacles (Elżbieta Kaczor-Urbanowicz, 2019; Li et al., 2024; Chandrakala et al., 2024). For instance, the challenges of low concentration of analyte in saliva can be overcome with digital PCR, highly sensitive immunoassays, and novel biosensing platforms, which allow quantification of sub-picogram levels of compounds (Kaczor-Urbanowicz et al., 2017). The future studies must focus on and address three main existing challenges for successful implementation of saliva as a diagnostic tool in routine clinical practice. It includes (1) rigorous standardization of analytical methods, (2) validation of biomarker controls in different populations, and (3) developing optimized protocols for saliva collection (Li et al., 2024). In summary, the non-invasive salivary diagnostic approach holds immense potential to emerge as a reliable tool for rapid and accurate detection of GI cancers in near future, provided that technical, biological, and clinical limitations are systematically addressed.

Multi-omics, integrating entire data pools of genomics, transcriptomics, proteomics, metabolomics, lipidomics and microbiomics, is regarded as one of the most promising approaches in translational cancer research, with regard to early diagnosis, tumor classification, prognostic, predictive phenotypes and others (Alyass et al., 2015; Liu et al., 2025; Koo et al., 2025; Dong et al., 2025). The integrative facet of multi-omics provides what the traditional single-omics analyses, otherwise useful in elucidating specific molecular mechanisms, cannot - that is the overall synergy of interactions at multiple levels between the host, tumor, and microbiome in the case of gastrointestinal cancers (Liu et al., 2025). As an example, while genomics studies have demonstrated the involvement of Adenomatous polyposis coli (APC) as a tumor suppressor gene highly mutated in colorectal cancers (CRC) (Zhang and Shay, 2017), and transcriptomics revealed the altered expression of proliferation-related pathways such as the WNT pathway hyperactivation (Kirov et al., 2025), it is integrative multi-omics that enables the linking of tumor genetic alterations with host immune responses, microbial dysbiosis, such as the enrichment of Fusobacterium nucleatum, and metabolic changes, including alterations in short-chain fatty acid production (Kim et al., 2025). A systematic research has recently demonstrated multi-omics methods incorporating AI analytics outperform traditional biomarkers (e.g., carcinoembryonic antigen and carbohydrate antigen 19-9) in detecting early stage GI cancers, but also contribute to personalized therapeutic strategies and immunotherapy optimization by allowing organoid models and microbiota profiling (Koo et al., 2025). Other clear advantages of multi-omics is that its “technologies” – the numerous –omics, above mentioned, are less or noninvasive approaches for diagnosing colorectal cancers (Ullah et al., 2022; Menyhárt and Győrffy, 2021), for identifying cancer subtypes, or disease mechanisms (Koo et al., 2025). The latest AI-models and machine learning (ML) algorithms, particularly deep learning and neural networks, are increasingly applied for analyzing the complex and very vast volumes of multi-omics data (Ali, 2023). An essential feature of these new ML models is their enabling building complex biological virtual interactions based on extracted data to reveal disease specific mechanisms (Debbadi and Boateng, 2025). This new efficient integration of muti-omics data has significantly benefited precision oncology domains such as cancer subtyping, genetic mutation prediction, risk group stratification, prognostication, outcome prediction, and clinical decision support, nicely detailed by Lise Wei et al. (2023). AI-assisted saliva liquid biopsy platforms also showed satisfactory accuracies in biomarker selection from abundant multi-omics data and determining the outcomes of different oral and maxillofacial disease, such as dental caries, oral cancer, and periodontal disease (Adeoye and Su, 2024). The automating evaluation of salivary gland biopsies has significantly benefited from ML models not only by increased detecting of glandular abnormalities, minimizing observer variability, but also by unifying multiple visual and molecular data towards a personalized diagnostics (Rawat et al., 2025).

Evidently, integration of AI-based platforms in multi-omics is changing from an innovation to a necessary technique for rapid data analysis, for the precise selection of salivary biomarkers, and integration of these with other clinical data of patients. At this stage, however, this accurate, non-invasive tool intended for diagnosis and patient-care has yet to be retrained and retested through large validation studies, under standardized protocols before any clinical implementation.

Some important limitations arise however concerning the current state of researches on the interactions between oral microbiota, salivary alterations, and gastrointestinal carcinogenesis. Importantly, majority of studies are either cross-sectional or case-control studies, which does not allow the precise causality between oral dysbiosis and tumoral manifestations. Longitudinal cohort studies should provide a clearer image on the role of oral dysbiosis in cancer.

With regard to Helicobacter pylori in oral or gastrointestinal carcinogenesis, current clinical and experimental evidence supports only a speculative interpretation of its potential synergistic interactions with other oncogenic oral bacteria (Wang et al., 2024a; Gusmaulemova et al., 2025). A further limitation across existing studies is the lack of methodological standardization, including heterogeneity in study design, sample types, and microbial analysis platforms, which complicates cross-study comparisons and interpretation of findings (Teles et al., 2020; Wang et al., 2024a, 2024b). There are also inconsistencies concerning the microbiota gut signatures, related either too often-reported overlapping microbial signatures (An et al., 2025) or to the automated platforms. Recently, a study analyzing previously-reported strong correlations between the DNA signatures of microbial organisms and different cancer types, found multiple errors in the genome database and the associated computational methods, flaws which invalidated the results leading to millions of false-positive findings (Gihawi et al., 2023).

Another significant disparity is the insufficient integration of functional and mechanistic data. While taxonomic associations are well-characterized, there is a pressing need to apply multi-omics approaches (Shaffer et al., 2017; Salihoglu, 2025). These should combine metagenomics, transcriptomics, metabolomics, and immune profiling to help us understand how microbial activity contributes to oncogenic signaling and metabolic changes (Shaffer et al., 2017; Salihoglu, 2025). In addition, preliminary studies suggest that oncogenic effects may be mediated by specific bacterial strains or virulence factors, rather than entire species, highlighting the need for higher resolution analyses that go beyond genus or species level classification (Li et al., 2025).

Saliva-based diagnostics have been identified as a promising non-invasive screening and monitoring tool (Zhao et al., 2025; Li et al., 2024). However, from a translational perspective, their clinical translation faces several challenges, like sampling protocols variability, lack of standardized collection and processing methods, limited large-scale validation studies, and salivary biomarkers instability after collection (Zhao et al., 2025; Li et al., 2024; d’Amone et al., 2021). Salivary diagnostics is emerging as a reliable clinical diagnostic tool, however future research is required to solve the lack of standardized protocols and validation challenges.

Recent progress in digital PCR, next-generation sequencing, biosensor methods, and AI-based data integration may help address current limits in analytical performance (Constantin et al., 2025). Ongoing and future longitudinal studies that combine multi-omics data with standardized methods are needed to test causal links, sharpen diagnostic specificity, and support the routine clinical use of saliva-based tests in gastrointestinal oncology (Koo et al., 2025).

## 7 Conclusion

Profiling of oral microflora for investigating GI oncology is a transformative approach in cancer diagnostics. Several studies have strongly supported the negative impact of oral dysbiosis in GI health, and proposed mechanisms through which opportunistic and pathogenic oral bacteria mediates tumorogenesis and carcinogenesis after being translocated to the GI mucosa. Hence, the microbial and molecular characteristics of saliva may be the key to decoding earliest perturbations leading to carcinogenesis. Certain bacterial shifts, such as decreases in commensals, like *Neisseria elongata* and *Streptococcus mitis*, alongside enrichment of opportunistic taxa including *Granulicatella adiacens* are distinctly associated with initiation of GI cancers. Similarly, *Leptotrichia* and *Fusobacterium nucleatum* are associated with oral microbial dysbiosis and tumor development (**Table 1**). Besides, salivary ctDNA, miRNA, EVs, and proteomic signatures have established connections between oral and systemic health, and indicated complex host-microbe interactions in the oral-gut axis. Overall, saliva is not just a simple digestive fluid but also a dynamic microbial and molecular information reservoir that has the potential to emerge as a non-invasive diagnostic tool for GI cancer detection in near future.
